# Oxidative Stress and Adipocyte Biology: Focus on the Role of AGEs

**DOI:** 10.1155/2015/534873

**Published:** 2015-03-23

**Authors:** Florence Boyer, Jennifer Baraka Vidot, Alexis Guerin Dubourg, Philippe Rondeau, M. Faadiel Essop, Emmanuel Bourdon

**Affiliations:** ^1^UMR DéTROI, Inserm U1188 Diabète Athérothrombose Thérapies Réunion Océan Indien, Université de La Réunion, Plateforme CYROI, Saint Denis de La Réunion, France; ^2^Cardio-Metabolic Research Group (CMRG), Department of Physiological Sciences, Stellenbosch University, Stellenbosch, South Africa

## Abstract

Diabetes is a major health problem that is usually associated with obesity, together with hyperglycemia and increased advanced glycation endproducts (AGEs) formation. Elevated AGEs elicit severe downstream consequences via their binding to receptors of AGEs (RAGE). This includes oxidative stress and oxidative modifications of biological compounds together with heightened inflammation. For example, albumin (major circulating protein) undergoes increased glycoxidation with diabetes and may represent an important biomarker for monitoring diabetic pathophysiology. Despite the central role of adipose tissue in many physiologic/pathologic processes, recognition of the effects of greater AGEs formation in this tissue is quite recent within the obesity/diabetes context. This review provides a brief background of AGEs formation and adipose tissue biology and thereafter discusses the impact of AGEs-adipocyte interactions in pathology progression. Novel data are included showing how AGEs (especially glycated albumin) may be involved in hyperglycemia-induced oxidative damage in adipocytes and its potential links to diabetes progression.

## 1. Introduction

Diabetes and associated pathologies are major health problems with an existing high and continuously rising prevalence worldwide. For example, more than 55 million individuals are burdened with this condition in Europe with it being projected to further increase to 64 million by 2030 [1]. Diabetes is a multifaceted disorder that is characterized by various metabolic derangements, with hyperglycemia as a major culprit. It is also associated with severe complications; for example, diabetes doubles the risk of developing cardiovascular diseases (CVD) that currently constitute the leading cause of mortality in developed countries [2]. Poor lifestyle choices are strongly connected to diabetes development, with especially suboptimal dietary intake and the lack of exercise linked to obesity onset. The latter usually includes excessive fat accumulation in adipose tissues, with such persons exhibiting relatively high body mass index (BMI) values of typically greater than 30 kg/m^2^. However, obesity* per se* does not represent an underlying medical condition but rather associated complications such as insulin resistance, type 2 diabetes, and CVD [3]. For example, obese persons with a BMI equal to 30 kg/m^2^ display a tenfold increase in risk for developing obesity-related pathologies compared to normal weight persons [4]. Together these studies demonstrate that the tremendous increase in obesity and associated pathologies (such as diabetes) constitute a significant global burden of disease that requires serious intervention strategies to counter its growing threat. In addition, a greater understanding of underlying mechanisms linking obesity to associated pathologies is essential as it may lead to the development of novel therapeutic interventions.

Oxidative stress, and more specifically oxidative damage to proteins, is increasingly thought to play a central, mechanistic role in this context as it is associated with modifications in the activities of biological compounds and cellular processes that may be linked to pathological complications. In support, the pathophysiologic perturbations connected with obesity-related diabetes are robustly associated with hyperglycemia-induced oxidative stress [5, 6]. Here oxidative stress may originate from various sources, with the mitochondrion proposed to play a major role as what was previously shown by our laboratory for the heart [7]. Furthermore, our recent data demonstrate that extra-mitochondrial sources such as NADPH oxidases can also generate reactive oxygen species (ROS) in cardiomyoblasts exposed to simulated hyperglycemic conditions [8]. Such oxidative stress is further fueled by excessive ROS production from glucose autoxidation and also the nonenzymatic, covalent attachment of glucose molecules to circulating proteins that results in the formation of glycated proteins and advanced glycation endproducts (AGEs) [9]. Greater AGE availability can in turn lead to downstream consequences, that is, binding to the receptor for AGE (RAGE) on target cells that induces several intracellular phenomena that likely contribute to the onset of diabetic complications (recent review in [10]). Higher systemic glucose levels can therefore lead to modifications of target proteins with severe downstream effects. For example, enhanced glycation of albumin (major protein in circulation) with diabetes significantly impairs its normal antioxidant function, while at the same time it also acquires additional detrimental properties [11, 12].

Despite the pivotal part that adipocytes play in the onset of several physiological/pathological processes, the role of increased AGEs formation in such tissues is not well understood and it is considered a slowly emerging research niche area [13]. For example, the first studies showing the impact of AGE-modified bovine serum albumin (BSA) on adipocytes were only published in 2003 [14, 15]. This minireview will therefore focus on the impact of AGEs-adipocyte interactions in terms of diabetes pathology progression. The background of AGEs formation and adipose tissue biology will initially be reviewed and thereafter the focus will shift to the link between AGEs and adipocytes. We will also include recent data focusing on glycated albumin and its link to hyperglycemia-induced oxidative damage in adipocytes.

## 2. AGEs Formation and Receptors

**Figure 1:**
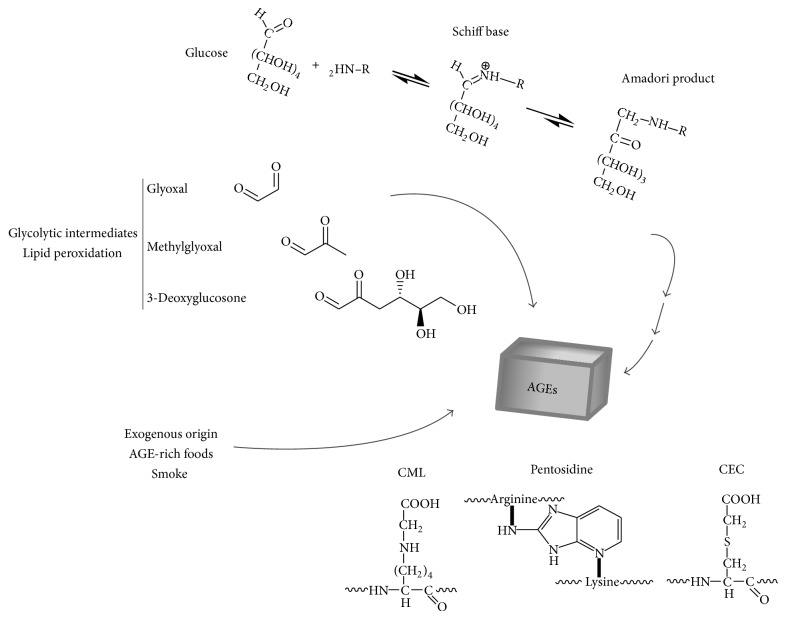
*Formation of AGEs.* AGEs can have different origins such as rearrangements of Amadori products, the latter arising from the glycation reaction. It can also be formed from glycolytic products with an exogenous origin. The glycation reaction mainly affects lysine, arginine, and cysteine residues leading to the formation of the following major AGEs: N^*ε*^-(carboxymethyl)lysine (CML), the crosslinker pentosidine, and S-(carboxymethyl)cysteine (CEC) (adapted from [10, 12, 13, 18–22]).

Several reaction cascades can result in AGEs formation, with the steps leading to glycation known as the Maillard reaction that was discovered by the famous French chemist Louis Camille Maillard during the early 1900s. This results in the nonenzymatic and nonoxidative covalent attachment of glucose molecules to target proteins, lipids, and nucleic acids [16]. Glycoxidation refers to the radical-mediated oxidation reaction of both free and protein-bound sugars [2]. The Amadori rearrangement of glycated proteins gives rise to advanced glycoxidation endproducts (also termed AGEs) [17]. The high variety in such reactions renders AGEs a heterogeneous group of chemically modified proteins [13]. For example, if lysine residues are particularly prone to glycation, the reaction can also affect arginine and cysteine residues leading to the generation of the major AGEs, that is, N^*ε*^-(carboxymethyl)lysine (CML), the crosslinker pentosidine, and S-(carboxymethyl)cysteine (CEC) [12] (Figure 1).

AGEs formation can result both from exogenous sources (dietary intake) and also due to high glucose availability that can trigger the Maillard reaction. AGEs were first identified in food processing technology and used to improve its quality in terms of taste, texture, and sensorial properties. However, recent studies by the Vlassara laboratory revealed the importance of dietary-related AGEs in the development of metabolic disorders and uncovered a novel paradigm; that is, AGEs can precede the onset of diabetes mellitus [18]. Interestingly, other exogenous AGEs sources have also been identified; for example, it is found in cigarette smoke and as a result smokers exhibit relatively high systemic levels [25].

What about AGEs formation within the* in vivo* context? Such modifications occur on a much longer time scale compared to exogenously supplied AGEs found in various dietary and other sources. Thus* in vivo* glycation mainly affects circulating proteins with a relatively long half-life such as albumin, the most abundant plasma protein [12]. It is also well established that enhanced albumin glycation with diabetes is associated with the early occurrence of vascular complications, together with functional protein alterations [12, 26–28]. However, AGEs formation and accumulation in diabetic individuals can result from various biochemical reactions, that is, “many roads leading to Rome” [29]. Here it can result from the reaction with highly reactive glucose-derived glycolytic intermediates such as glyoxal, methylglyoxal, or 3-deoxyglucosone that react 7- to 200-fold faster than glucose [19]. Of note, such AGEs are not formed solely from carbohydrate metabolism but can also result from lipid oxidation and degradation [20].

**Figure 2:**
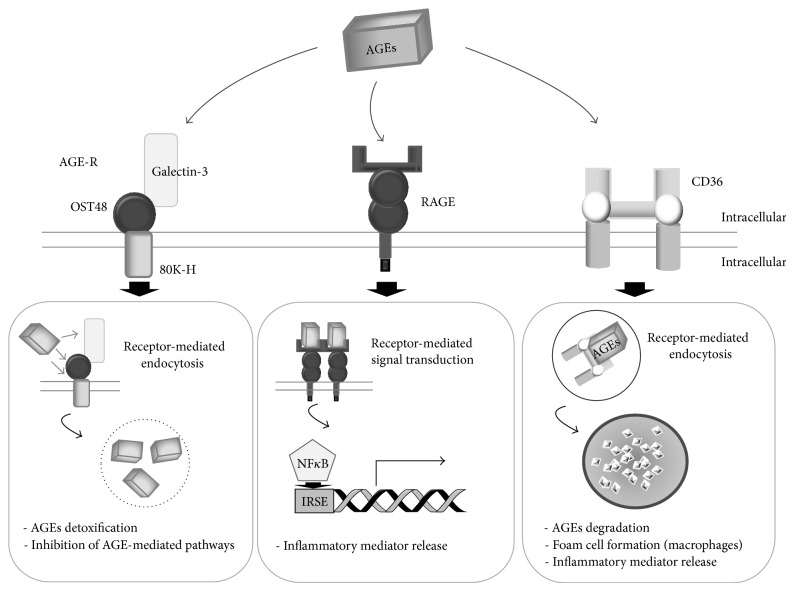
*Different types of AGEs receptors.* Three major AGEs-receptor pathways are represented: the AGE-R complex constituted by (a) AGE-R1 80K-H, AGE-R2 OST-48, and AGE-R3 galectin-3, (b) RAGE, and (c) CD36 that belongs to SR-B (macrophage scavenger receptor type B) (adapted from [10, 12, 23, 24]).

The glycation of plasma proteins leads to several downstream consequences and this is initiated by its binding to cell membrane-specific receptors. The different receptors able to recognize AGEs can be schematically divided into three types depending on the signaling pathways subsequently induced (Figure 2). RAGE represents the most studied receptor and is mainly expressed on vascular, endothelial, and smooth muscle cells and on monocyte/macrophage membranes [10, 30]. The RAGE family also includes the AGE-R complex constituted by AGE-R1 80K-H, AGE-R2 OST-48, AGE-R3 galectin-3, SR-A (macrophage scavenger receptor types I and II), and SR-B (SR-B type I and CD36) [23, 30]. Several physiologic and pathophysiologic roles have been reported for receptors able to bind to AGEs [10]. Most are considered as toxic effects, that is, downstream oxidative stress generation and the initiation of inflammatory cascades such as ROS-related activation of the proinflammatory transcriptional modulator, NF*κ*B [10]. Conversely, AGE-R1 and R3 compete with RAGE for AGE binding with resultant opposing effects such as the protective role of AGER against ROS formation, oxidative stress, and increasing AGE degradation and urinary excretion [18]. Scavenger receptors are a family of receptors able to recognize oxidized or acetylated low density lipoproteins at macrophage surfaces. Scavenger receptors A and B (CD36 and SR-B, resp.) are involved in the intracellular accumulation of cholesterol and the formation of foam cells from macrophages in the early state of atherosclerosis [31].

As discussed in this section, AGEs have different origins and several receptors play crucial roles in mediating their downstream intracellular effects. In this regard several studies focused on the effects of AGEs on vascular cells and also circulating cells such as monocytes or circulation-derived cells. However, despite the important role of adipocytes in the etiology of diabetes, little is known about the effect of AGEs on such cells. Thus in the following sections an overview of adipose tissue biology and the impact of AGEs on adipocytes will be discussed.

## 3. Overview of Adipose Tissue Biology

Obesity is defined as a condition characterized by excessive fat accumulation and storage [32]. Hence, with caloric abundance, fat is stored as triglycerides (TG) in adipocytes within adipose depots. However, fat stores can also be utilized during times of caloric debt to provide energy substrates by the release of nonesterified fatty acids (NEFA) into circulation [33]. With obesity there is a higher risk for the onset of cardiometabolic diseases and here increased adiposity (especially visceral adiposity) is linked with a greater risk for insulin resistance/type 2 diabetes [34–36]. Although the link(s) between obesity, insulin resistance, and the development of type 2 diabetes is still not fully elucidated [33], several studies show that the dysregulation of adipocyte function is a crucial role player associated with diabetes pathology progression. It is therefore not surprising that numerous investigations are focusing on this problem in order to derive novel therapies that target adipocyte dysregulation within the diabetic milieu [37–39].

The underlying biology of adipose tissue is far more complex than the original concept of its sole function being that of a fat storage depot [40, 41]. In addition to TG storage and NEFA release into circulation, adipocytes express and secrete a variety of active biomolecules or “adipokines” that regulate many physiologic processes such as insulin sensitivity, appetite, immunity, and reproduction [42, 43]. Although various processes are implicated in the development of insulin resistance in adipocytes, inflammation and oxidative stress emerge as robust causative factors in this instance [6, 34, 44–46]. In support, a growing number of publications highlight the role of inflammation and oxidative stress and its impact on adipocyte disorders; for example, recent work found increased NF*κ*B-mediated cytokine release from adipocytes isolated from obese individuals [47]. Growth hormone administration also improved glucose intolerance in obese mice presumably by decreasing adipose mass, oxidative stress, and chronic inflammation in visceral fat stores [48]. Moreover, glutathionylated lipid aldehydes, identified as products of adipocyte oxidative stress, result in the activation of macrophage inflammation [49], while an antioxidant molecule attenuated hypoxia-induced oxidative stress, inflammation, and mitochondrial dysfunction in 3T3-L1 adipocytes [50].

Among the cytokines originating from adipocytes, secretion of the S100 calcium binding protein B, a RAGE ligand, was recently shown to be enhanced in 3T3 L1 cells incubated under inflammatory conditions and triggered macrophage activation though RAGE [51].

Together these studies demonstrate that oxidative stress and inflammation are crucial pathophysiologic mediators that contribute to adipocyte dysregulation and the onset of various cardiometabolic complications.

Most studies implicating oxidative stress and inflammation in adipocyte pathophysiology employed* in vitro*-based methodologies with cells exposed to various stressors such as hypoxia, lipopolysaccharides, hydrogen peroxide, and hyperglycemic treatments [52–55]. Thus limited studies have examined the role of AGEs* per se* in adipocytes, with the detrimental effects of AGEs generally investigated in vascular cells and also circulating cells such as macrophages. The following section will therefore focus on recent, novel data regarding the impact of circulating AGEs on adipocytes.

## 4. AGEs Adipocyte Interactions and the Onset of Pathologies

As discussed earlier, the effects of AGEs on adipocyte function are limited; for example, a PubMed search with the terms “AGEs adipocytes glycation” retrieved only 19 references. What then is known about the effects of AGEs on adipocytes? Studies done thus far have identified the major downstream effects as a heightened inflammatory response as well as the generation of intracellular oxidative stress. For example, a recent study showed that AGEs augmented the expression of the prothrombotic/inflammatory regulator, plasminogen activator inhibitor-1, in rat white adipocytes, by a ROS-dependent pathway [56]. Moreover, glycated BSA increased the adipogenic potential of senescent preadipocytes (*in vitro* and* ex vivo*) via the AGEs-RAGE axis together with an impairment of p53 function [57]. Here this occurs by direct binding of RAGE to cytosolic p53 together with the AGEs-RAGE suppression of p53 transcript levels. This in turn enhances the adipogenic potential of preadipocytes, with detrimental long-term effects. Recent experiments performed in our laboratory demonstrated that glycated albumin exposure induced oxidative stress in primary human adipocytes thereby leading to the accumulation of oxidized proteins [58, 59]. A proteomic-based approach allowed us to also determine preferential protein carbonylation targets in human mature adipocytes treated with glycated versus native albumin [59]. Our studies also revealed greater insights into some of the underlying mechanisms as AGEs-treated adipocytes displayed decreased ubiquitin proteasomal system (UPS) activities and were therefore unable to clear damaged proteins. These data therefore suggest that the origin of accumulated oxidized proteins in AGEs-treated adipocytes likely stems from increased intracellular ROS production together with an impaired UPS [59, 60].

What about the receptors for AGE found on adipocyte membranes? Kuniyasu et al. (2003) were the first to discover the presence of CD36 on mouse adipocyte (3T3 L1 cell lines) cell membranes and on human adipocytes from primary cultures that were able to bind and facilitate AGEs endocytosis and degradation [15]. Two years later the same group identified a pathological role for CD36 in AGE binding in adipocytes, that is, resulting in decreased* in vivo* leptin expression and attenuated insulin sensitivity [61, 62]. In addition, adipocytes exposed to AGEs exhibited diminished adiponectin expression (usually associated with insulin resistance) [63]. AGEs exposure also caused impaired functionality of adiponectin and this may further contribute to the development of insulin resistance [64, 65].

**Figure 3:**
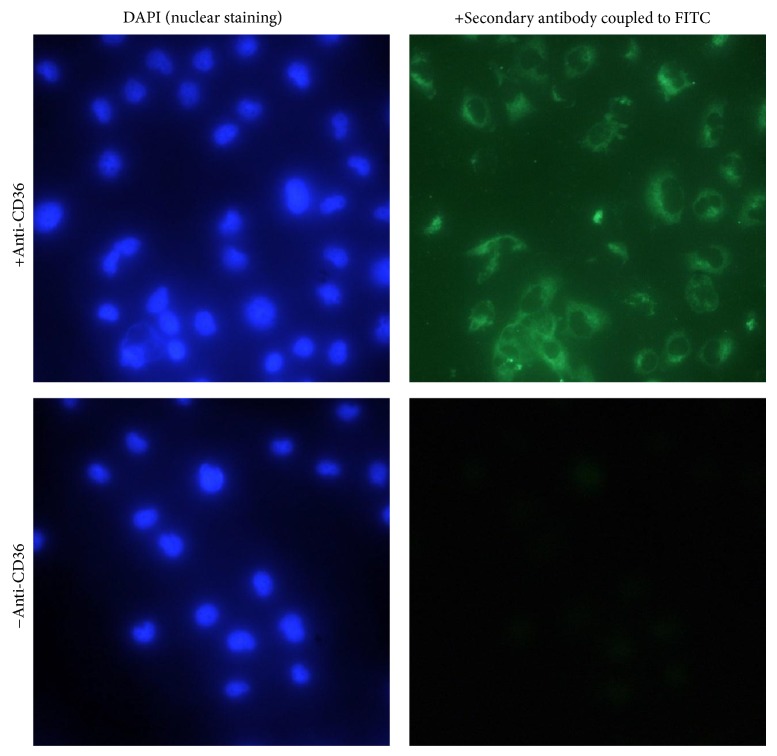
*Adipocyte cell line expresses CD36 receptor.* SW872 adipocytes were cultured on 12 mm diameter poly(L-lysine) coverslips until 80% confluency. Cells were fixed in diluted* para*formaldehyde solution (4% v/v in PBS) at room temperature for 20 min and stained successively with a primary human CD36 antibody (1 : 50 in 1% PBS/BSA) for one hr, followed by incubation with an Alexa Fluor 488 goat anti-rabbit IgG antibody (1 : 1,000) for 1 hr and with DAPI solution (1 : 1,000) for 10 min (for staining of the nucleus). Cells were washed 3x in 1% PBS/BSA between each step of different incubation periods. Cells were imaged using a Nikon eclipse microscope and NIS-Element software (Nikon Corporation, Tokyo, Japan). ^###^ *P* < 0.001 (*vs.* HSA) by Student's unpaired *t* test (*n* = 3).

**Figure 4:**
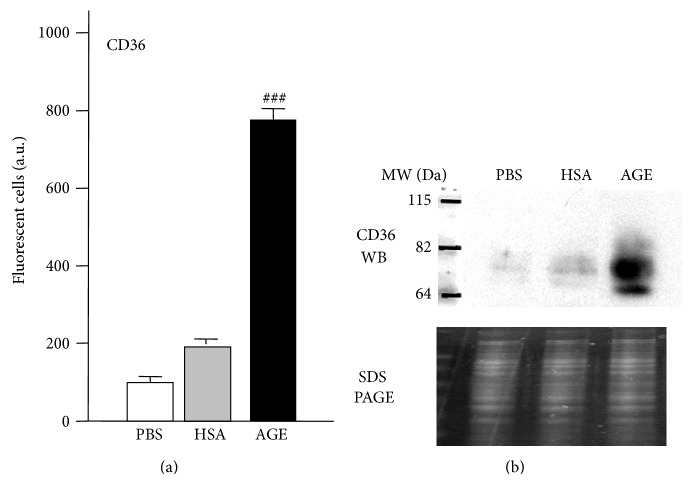
*Glycated albumin induces CD36 expression in SW872 adipocytes.* (a) SW872 adipocytes were incubated for 24 hr in the absence (PBS) or the presence of 50 *μ*M native human serum albumin (HSA) or AGEs constituted by methylglyoxal- (MGO) modified HSA. The relative quantification (% of fluorescent cells versus control PBS) of CD36 receptors was determined by employing a Becton Dickinson FACScan (BD Biosciences, San Jose, CA) after staining with a primary human CD36 antibody (1 : 50) for 1 hr, followed by incubation with the secondary Alexa Fluor 488 conjugated anti-rabbit (1 : 100) and PE-conjugated anti-mouse antibodies (1 : 100) for an additional hour. Cells were washed with a 1% PBS/BSA blocking buffer between each incubation step. (b) For the CD36 Western blot, 20 *μ*g proteins were isolated from SW872 cell lysates (with different treatments as indicated), separated by SDS-PAGE, and transferred onto a nitrocellulose membrane (Bio-Rad Laboratories, Hercules, CA) using a liquid transfer system. Membranes were soaked overnight with blocking buffer (PBS/0.1% Tween/1% BSA) and subsequently incubated in blocking buffer with a primary human CD36 antibody (1 : 200) for 2 hr, followed by incubation with a secondary HRP-conjugated sheep anti-mouse IgG antibody (1 : 2,000). Membranes were washed with a blocking buffer (0.1% PBS/1% Tween) between different incubation steps. Protein bands were detected by standard ECL methods (Amersham Biosciences, Amersham, UK) and visualized with a Kodak 2000R Image station (Eastman Kodak, Rochester, NY), and routine densitometric analysis was performed for quantification.

The SW872 cell line has only recently been employed as an adipocyte cell model [59, 66–68] and has the advantages of a human origin and also does not require any incubation cocktails to differentiate it into mature adipocytes [66]. We therefore initiated studies using this cell line and found elevated oxidative stress and protein damage in AGEs-treated SW872 adipocytes. Such damaging effects could be blunted with the co-treatment of nutritional antioxidants thereby further implicating oxidative stress in this process [60, 69, 70]. Unpublished findings from our group also demonstrate, for the first time as far as we are aware, that CD36 is expressed in the SW872 cell line (Figure 3). Furthermore, fluorescence-activated cell sorting (FACS) and Western blot data show increased CD36 expression in SW872 adipocytes exposed to glycated albumin purified from diabetic persons (Figure 4). However, as there are no data regarding CD36 expression in adipocytes within the diabetic context, further research is needed. This is currently being pursued by our laboratory in order to assess the correlation between AGEs levels and CD36 expression in adipose tissues isolated from transgenic diabetic mice (Db/Db strain).

## 5. Conclusions

Enhanced AGEs formation and the subsequent tissue and cellular oxidative damage, together with inflammation, are now well established in pathophysiologic disorder progression. Increasing evidence shows that AGEs accumulation in adipose tissue may contribute to obesity-associated insulin resistance. However, the precise nature and mechanisms of AGEs impact on the adipocyte's function are only now slowly emerging and remain poorly understood. The picture is also more complex than what was covered in this minireview, focusing only on the role of circulating AGEs versus intracellular AGEs in adipocytes. Moreover, the human body's defense mechanisms to counter the overproduction of AGEs were not discussed in this paper. This field is a “hot topic” and here dysregulation of the glyoxylase system, constituted by glyoxylase-1 (GLO1) and glyoxylase II (GLO2), is emerging as important contributors to higher AGEs levels [13]. For example, a recent study established that GLO1 overexpression attenuated AGEs and diminished oxidative stress [71]. Thus further research is required to gain greater insights into the precise nature of the AGEs-RAGE axis in adipocytes and its relevance within the diabetic milieu, with the focus also on extra- and intracellular AGEs availability and the glyoxylase defense system. Such novel insights may in turn unlock novel pharmaceutical and/or nutritional strategic developments that should help blunt obesity-related insulin resistance progression.

## List of Abbreviations

AGE: Advanced glycation endproduct
BMI: Body mass index
CEC: Carboxymethyl-cysteine
CML: Carboxymethyl-lysine
CVD: Cardiovascular diseases
FACS: Fluorescence-activated cell sorting
GLO: Glyoxylase
NEFA: Nonesterified fatty acids
RAGE: Receptor for AGE
ROS: Reactive oxygen species
TG: Triglycerides.
